# Low Bone Turnover and Low BMD in Down Syndrome: Effect of Intermittent PTH Treatment

**DOI:** 10.1371/journal.pone.0042967

**Published:** 2012-08-14

**Authors:** Tristan W. Fowler, Kent D. McKelvey, Nisreen S. Akel, Jaclyn Vander Schilden, Anthony W. Bacon, John W. Bracey, Timothy Sowder, Robert A. Skinner, Frances L. Swain, William R. Hogue, Donna B. Leblanc, Dana Gaddy, Galen R. Wenger, Larry J. Suva

**Affiliations:** 1 Department of Physiology & Biophysics, University of Arkansas for Medical Sciences, Little Rock, Arkansas, United States of America; 2 Department of Orthopaedic Surgery, Center for Orthopaedic Research, University of Arkansas for Medical Sciences, Little Rock, Arkansas, United States of America; 3 Department of Genetics, University of Arkansas for Medical Sciences, Little Rock, Arkansas, United States of America; 4 Department of Pharmacology, University of Arkansas for Medical Sciences, Little Rock, Arkansas, United States of America; Inserm U606 and University Paris Diderot, France

## Abstract

Trisomy 21 affects virtually every organ system and results in the complex clinical presentation of Down syndrome (DS). Patterns of differences are now being recognized as patients’ age and these patterns bring about new opportunities for disease prevention and treatment. Low bone mineral density (BMD) has been reported in many studies of males and females with DS yet the specific effects of trisomy 21 on the skeleton remain poorly defined. Therefore we determined the bone phenotype and measured bone turnover markers in the murine DS model Ts65Dn. Male Ts65Dn DS mice are infertile and display a profound low bone mass phenotype that deteriorates with age. The low bone mass was correlated with significantly decreased osteoblast and osteoclast development, decreased bone biochemical markers, a diminished bone formation rate and reduced mechanical strength. The low bone mass observed in 3 month old Ts65Dn mice was significantly increased after 4 weeks of intermittent PTH treatment. These studies provide novel insight into the cause of the profound bone fragility in DS and identify PTH as a potential anabolic agent in the adult low bone mass DS population.

## Introduction

Although Down syndrome (DS) was initially described in 1866 [1], the pathophysiology of many clinical aspects of the DS phenotype have not been elucidated. Low bone mass and the associated increased fracture rates are clinical features that complicate DS [2]. As the life expectancy of individuals with DS has increased to greater than age 50 [3], [4], the bone health of adolescent and adult DS patients has become an important medical issue. With the increasing life expectancy, many concerns regarding the risk of osteoporosis have been raised [5], [6], [7]. In fact, the accrual of bone mass during childhood and adolescence may reduce osteoporosis risk later in life and low bone mass in young adulthood is a strong risk factor for later osteoporosis and fracture [8], [9].

Several investigators, including ourselves have reported that adults (and children) with DS have lower bone mass, expressed as BMD, especially in the lumbar spine, compared with their peers without mental retardation or with mental retardation but without DS [5], [10], [11], [12], [13]. Known secondary causes for low BMD include dietary insufficiency (vitamin D and calcium intake), endocrine (hypothyroidism, hyperparathyroidism, hypogonadism), and autoimmune disorders (celiac disease) which lead to inadequate nutrition. Low activity levels, low sunlight exposure and anti-convulsant use have also been associated with decreased bone mass but these are not consistent risk factors in DS, leaving the underlying pathophysiology unknown [2]. In addition, despite numerous reports in the literature of BMD measurements in DS, the measurement of bone biochemical markers in community dwelling DS patients compared to the normal population or the detailed analysis of the skeletons of DS mouse models are scarce; [7], [14]. Finally, no bone anabolic therapies have been evaluated in DS.

The Ts65Dn mouse is one of several mouse models of DS that has been developed to investigate the pathology of DS phenotypes [15]. The Ts65Dn mouse strain is a DS model characterized by segmental trisomy for the region of mouse chromosome (Mmu) 16 that contains approximately 75% of the human chromosome (Hsa) 21-homologous genes. Approximately 324 genes recognized on human chromosome 21 (Hsa21) are split over three mouse chromosomes: Mmu10, Mmu16 and Mmu17 [16].

To investigate bone health in DS, we determined the skeletal phenotype of the Ts(17^16^)65Dn (Ts65Dn) mouse model of DS at both 3 and 24 months of age [17], [18] and evaluated the efficacy of an anabolic regimen of human PTH in these animals. We hypothesized that in addition to the characteristic behavioral and craniofacial features of DS exhibited by Ts65Dn mice, the skeletons of these mice would model the bone phenotype of the human condition. In this study, the skeletal phenotype of young and aged Ts65Dn mice was determined. When compared with low BMD and bone turnover measurements in adult male DS patients [13], male Ts65Dn mice phenocopy the bone phenotype of the patients with striking consistency.

The studies revealed that just as in people with DS, the Ts65Dn mice have low bone mass that is the result of an intrinsic cellular defect resulting in low bone turnover and decreased bone formation. The low bone turnover state is present despite the profound hypogonadism and the low bone mass was increased by 4 weeks of intermittent PTH therapy. As such, anabolic treatment options should be considered in the management of the low bone mass that is prevalent in DS.

## Results

### Skeletal Phenotype of Trabecular and Cortical Bone in Ts65Dn Mice

**Figure 1:**
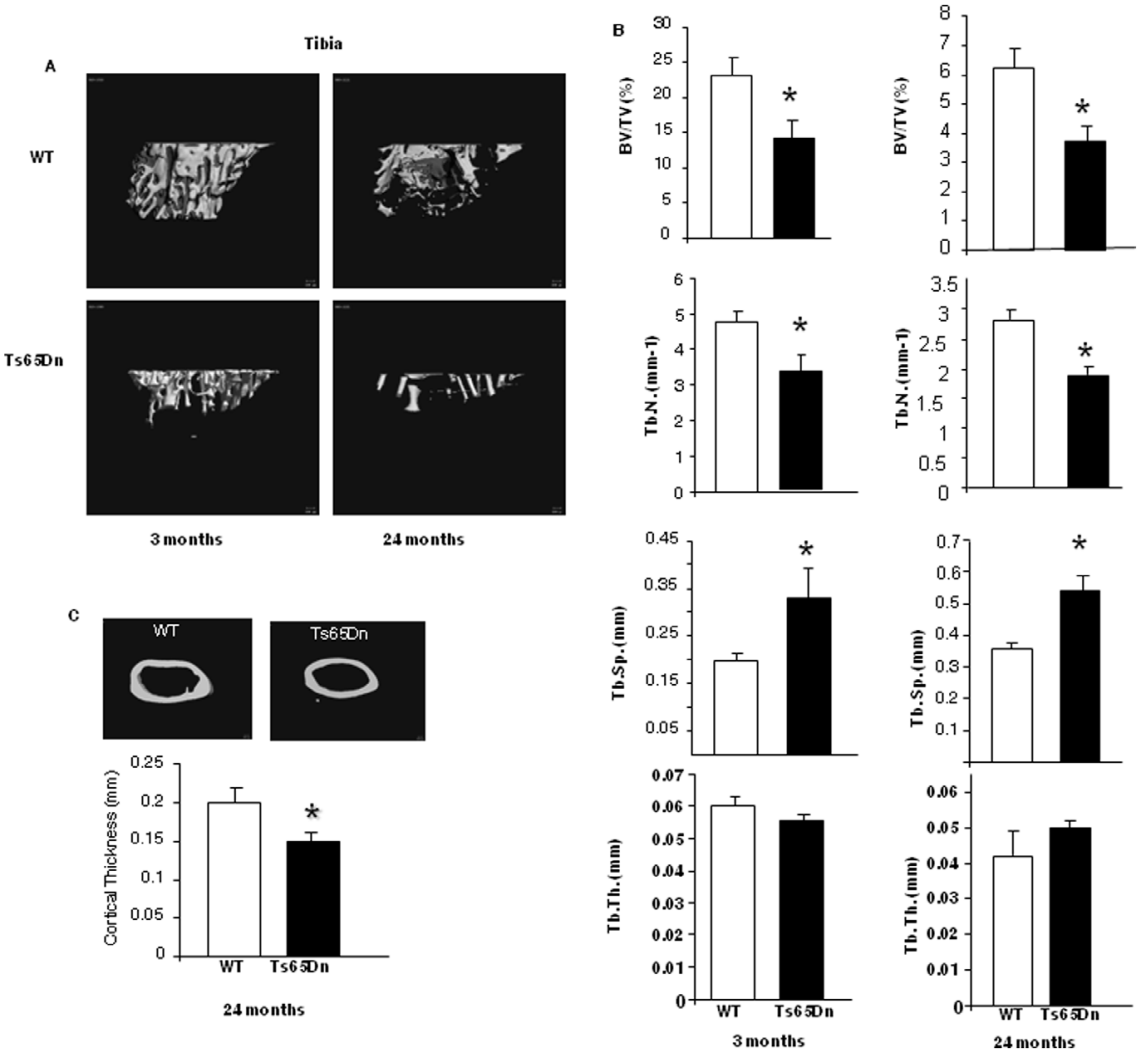
Trisomy effects on the proximal tibia and femur or 3-month and 24-month Ts65Dn mice. (**A**) Micro CT reconstructions of metaphyseal tibial cancellous bone in 3-month (left column) and 24-month (right column) old WT (top) and Ts65Dn (bottom) mice. The low bone volume is evident at 3-months in the Ts65Dn mice vs. WT. (**B**). Micro CT analysis of % bone volume/tissue volume (BV/TV), trabecular number (Tb.N.), trabecular thickness (Tb.Th.), trabecular separation (Tb.Sp) in Ts65Dn 3-month and 24-month old WT and Ts65Dn proximal tibia. (**C**). Decreased cortical thickness at the femoral midshaft by micro CT in 24 month old WT and Ts65Dn mice. Representative MicroCT reconstructions of the midshaft and measurement of cortical thickness in both WT and Ts65Dn are shown. *, p<0.05 vs. WT.

Three-dimensional analysis of trabecular microarchitecture by µCT was performed on the proximal tibia and distal femur. Ts65Dn mice had a significantly decreased bone volume/total volume (BV/TV) at both 3 months and 24 months of age (Figure 1A; Figure S1). There was also a significant decrease in the trabecular number (Tb.N.) in 3 month and 24 month old Ts65Dn mice (Figure 1B) and a corresponding significant increase in the trabecular spacing (Tb.Sp.) (Figure 1B). However, although Tb.N. was significantly decreased, trabecular thickness (Tb.Th.) was not altered in Ts65Dn mice at either age, suggesting that although bone turnover is decreased, the molecular regulation of trabecular thickness is maintained in these animals.

In addition to examining the effects of aneuploidy on trabecular bone, we also examined cortical bone from Ts65Dn mice (Figure 1C). Transverse CT slices were acquired at the femoral midshaft to assess total cross sectional area, cortical bone area, and medullary area (TA, BA, and MA, (mm^2^)); bone area fraction (BA/TA, %); cortical thickness (mm); anteroposterior and mediolateral diameters (mm). Sub-region analysis of the mid-shaft of the femur revealed that trisomy significantly decreased cortical thickness in both 3-month old (Figure S2) and 24-month Ts65Dn mice (Figure 1C). Cortical cross-sectional area (CSA), periosteal perimeter, and total cross-sectional area were also significantly decreased in the 24-month Ts65Dn mice compared to age-matched WT control mice (Figure S2), while cortical porosity was unchanged (Data not shown). The decrease in the cortical bone geometric parameters of 24-month old Ts65Dn mice was associated with significantly decreased mechanical strength. The peak load and stiffness of 24 month old Ts65Dn femurs was significantly decreased compared with age-matched littermate controls (Figure S2). Thus, the skeletal effects of trisomy in Ts65Dn mice are decreased trabecular bone volume and architecture, as well as changes in cortical parameters that result in decreased overall bone stiffness and bone strength.

### Decreased Bone Turnover is Responsible for the Low Bone Mass in Ts65Dn Mice

**Figure 2:**
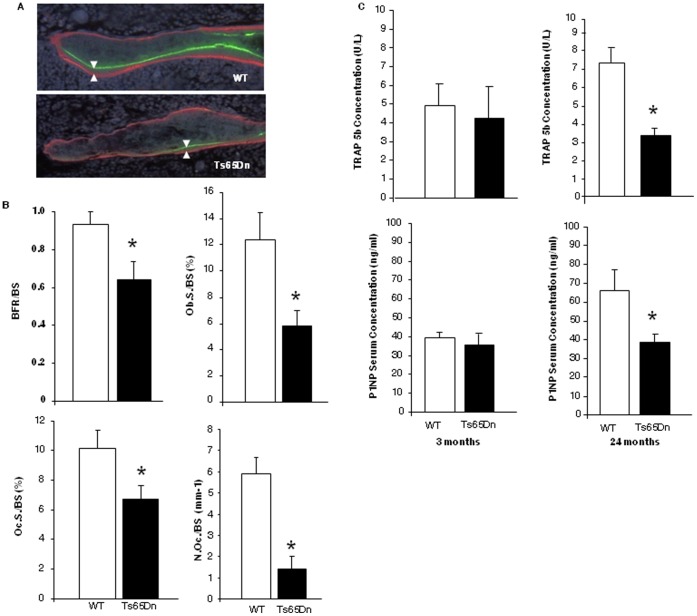
Bone Formation and bone turnover is decreased in Ts65Dn Mice. (**A**). Representative double-fluorochrome-labeled regions of trabecular bone in the proximal tibia of 3 month old WT (top) and Ts65Dn (bottom). *Arrowheads* indicate the distance between the two labels used to calculate the bone formation rate normalized to bone surface (BFR/BS) (um^3^/um^2^/day). (**B**). Tibial sections (N = 6 per group) were used to quantify BFR/BS, percent osteoblast surface/bone surface (Ob.S./BS), percent osteoclast surface/bone surface (Oc.S./BS) and number of osteoclasts/bone surface (N.Oc./BS.) (**C**). Serum obtained from WT and Ts65Dn mice at 3- and 24-months of age was analyzed for bone resorption, tartrate resistant acid phosphatase 5b (TRAP 5b) and bone formation, procollagen-1 N-terminal peptide (P1NP) markers by ELISA. *, p<0.05 vs. respective WT vehicle control.

Several quantitative analyses were performed to determine whether the decreased bone mass and strength in Ts65Dn mice were mediated by decreased bone turnover despite hypogonadism, as we have observed in people with DS [13]. Static histomorphometric analysis of the secondary spongiosa of paraffin-embedded sections of the decalcified proximal tibia (as well as femur; data not shown) of 3-month old mice (Figure 2) demonstrated that Ts65Dn mice have significant decreases in osteoblast surface per bone surface and osteoclast surface per bone surface, and significantly decreased numbers of osteoclasts on the bone surface. To determine if the observed decreases in osteoblast and osteoclasts were associated with decreased bone remodeling bone formation rate was measured using dynamic histomorphometry in non-decalcified methyl methacrylate-embedded sections as we have described [19], [20]. The analysis in 3-month old Ts65Dn mice demonstrated significantly decreased bone formation rate (BFR) per bone surface (Figure 2). The significantly decreased BFR resulted in significant changes in the serum concentration of markers for bone resorption and bone formation in 24-month, but not 3-month, old Ts65Dn mice. Serum TRAP 5b levels were significantly decreased indicating a decrease in overall bone resorption (Figure 2). Furthermore, there was a significant decrease in the serum P1NP levels, indicating a decrease in the amount of bone formation (Figure 2). These results demonstrate that the significantly decreased numbers of osteoblasts and osteoclasts on the bone surface resulting in decreased bone formation and bone resorption are responsible for the observed low bone mass phenotype in Ts65Dn mice. These observations recapitulate the low bone turnover phenotype in people with DS [13], confirm the reported low bone mass of Ts65Dn [14], and support the notion that DS patients with low BMD are candidates for anabolic but not anti-resorptive bone-targeted therapy.

### Cellular Defect in Ts65Dn Mice Involves Both Decreased Osteoblast and Osteoclast Differentiation

**Figure 3:**
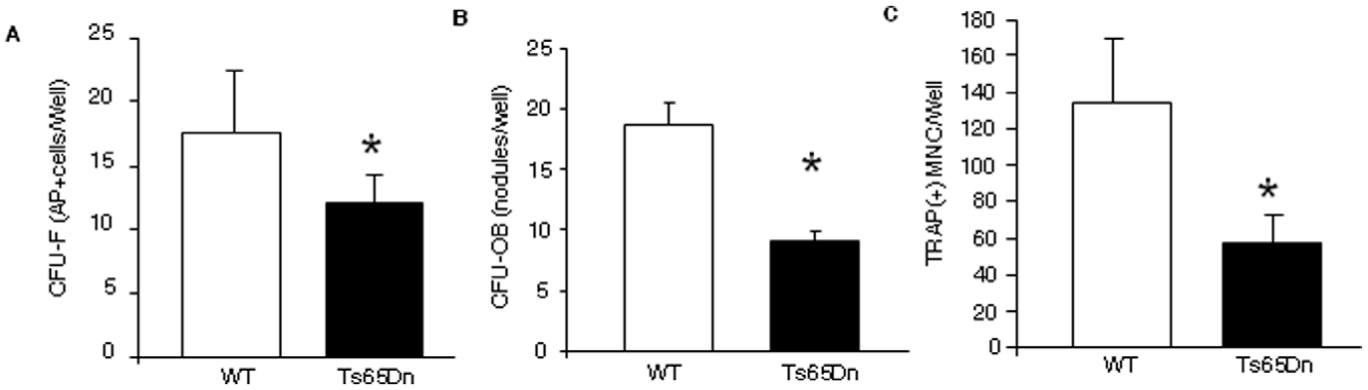
Osteoclast and osteoblast formation is significantly decreased in 3 month old Ts65Dn Mice. (**A**) *Ex vivo* recruitment into the osteoblast lineage was measured at culture day 10 by staining for alkaline phosphatase (AP) and counting the number of AP+ colony forming units (CFU-F) per well. (**B**) *Ex vivo* osteoblast differentiation was assessed at culture day 28 by staining mineralized bone nodules containing differentiated colonies of osteoblasts (CFU-OB) with Alizarin Red and the number of CFU-OB per well enumerated. (**C**) *Ex Vivo* osteoclast differentiation was assessed by staining on day 14 for tartrate resistant acid phosphatase (TRAP) and the number of TRAP+-multinucleated cells per well counted. *, p<0.05 vs. WT vehicle control.

In an effort to determine the cellular mechanism responsible for the low bone mass phenotype in Ts65Dn mice, *ex vivo* bone marrow cultures were initiated and stimulated towards either osteoblastogenesis or osteoclastogenesis [19], [21]. Bone marrow derived from Ts65Dn mice had a significantly decreased capacity for osteoblast recruitment compared to control littermates, as measured by the number of alkaline phosphatase (AP+) colonies per well after 10 days of culture in osteoblastogenic media (Figure 3A) [22]. Furthermore, the ability of these cells to undergo maturation to mineralizing osteoblasts was also significantly decreased when compared to littermate controls, as measured by the number of mineralized nodules per well (Figure 3B). Collectively, these results demonstrate that Ts65Dn mice have an intrinsic cell deficit in the mesenchymal lineage, leading to a decreased pool of cells that are capable of being recruited into the osteoblastic lineage.

*Ex vivo* bone marrow cultures from Ts65Dn and wild-type littermates were also cultured towards osteoclasts as described [19], [21]. After 14 days in culture, cells from the Ts65Dn bone marrow produced significantly fewer mature tartrate resistant acid phosphatase positive (TRAP+), multinucleated cells per well than wild type controls (Figure 3C). These data demonstrate that the hematopoietic lineage is also significantly affected in Ts65Dn mice, with a resulting decrease in osteoclastogenesis, paralleling the observed decrease in osteoclast surface/bone surface and activity observed *in vivo*. Therefore, the low bone mass observed in Ts65Dn is the result of a decrease in bone turnover, due to an intrinsic defect in the production of normal numbers of mature bone forming osteoblasts and bone resorbing osteoclasts. This cellular defect is occurring in the face of the hypogonadism and infertility that characterizes male Ts65Dn mice [2], [23].

### Intermittent PTH Increases BMD in Ts65Dn Mice

Given the low bone mass phenotype and low bone turnover states of both humans with DS and Ts65Dn mice, the effect of intermittent anabolic PTH therapy in 3 month old WT and Ts65Dn mice was determined. Intermittent PTH is the only FDA-approved bone anabolic therapy. Since the effects of PTH for the stimulation of bone mass in situations of low bone turnover are not well documented, two different doses of PTH(1–34) were used.

**Figure 4:**
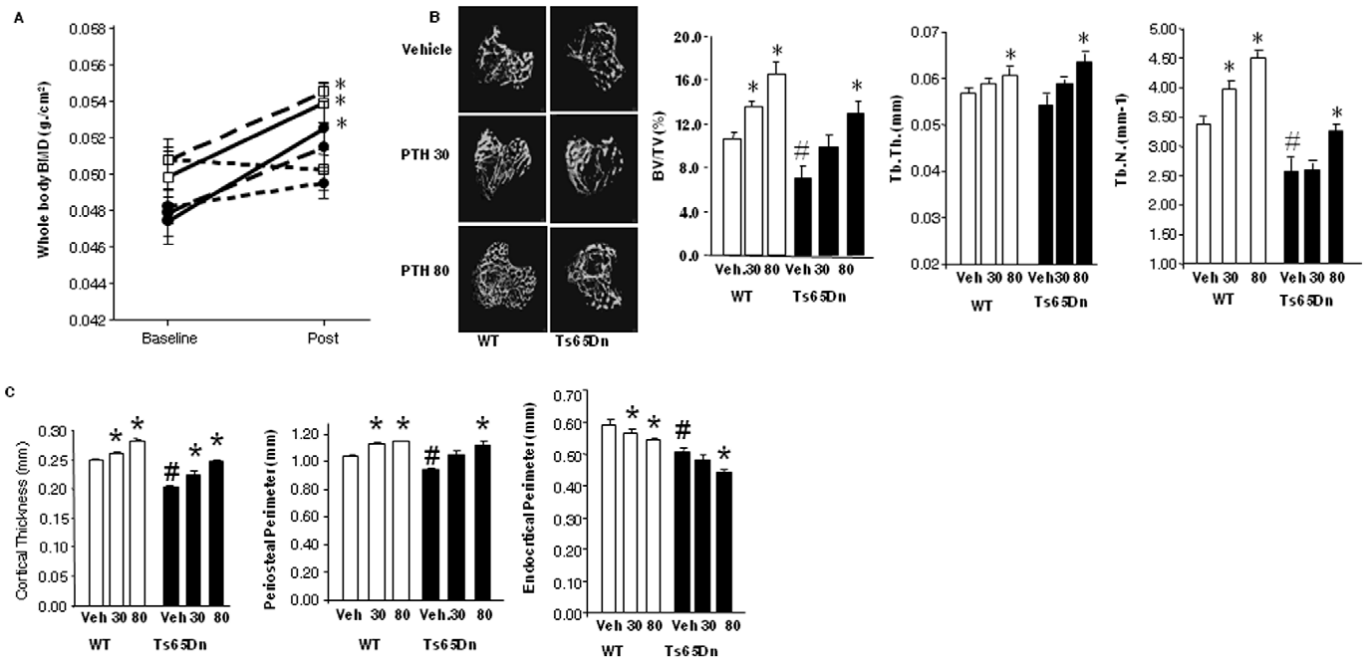
Efficacy of intermittent PTH in WT vs. Ts65Dn mice. Male mice (N = 6–8 per group) were given daily injections of vehicle or 30 or 80 ug/kg PTH(1–34) for 4 weeks. (**A**) BMD was measured at the beginning and end of the experiment. (open squares, WT; closed circles, Ts65Dn). Dotted lines show BMD of vehicle treated animals, dashed lines, 30 ug/kg PTH; solid lines, 80 ug/kg PTH (**B**) % bone volume/tissue volume (BV/TV), trabecular number (Tb.N.) and trabecular thickness (Tb.Th.) were determined in the tibia (open bars WT; solid bars Ts65Dn). Representative superior view of a transverse micro-CT images of the trabecular bone from the proximal tibia of representative animals in each group are shown. (**C**) Micro CT measurements of the effects of PTH treatment on cortical thickness, periosteal perimeter and endocortical perimeter in the distal tibia were performed (open bars WT; solid bars Ts65Dn). *, p<0.05 vs. respective vehicle control; #, p<0.05 vs. WT vehicle.

**Figure 5:**
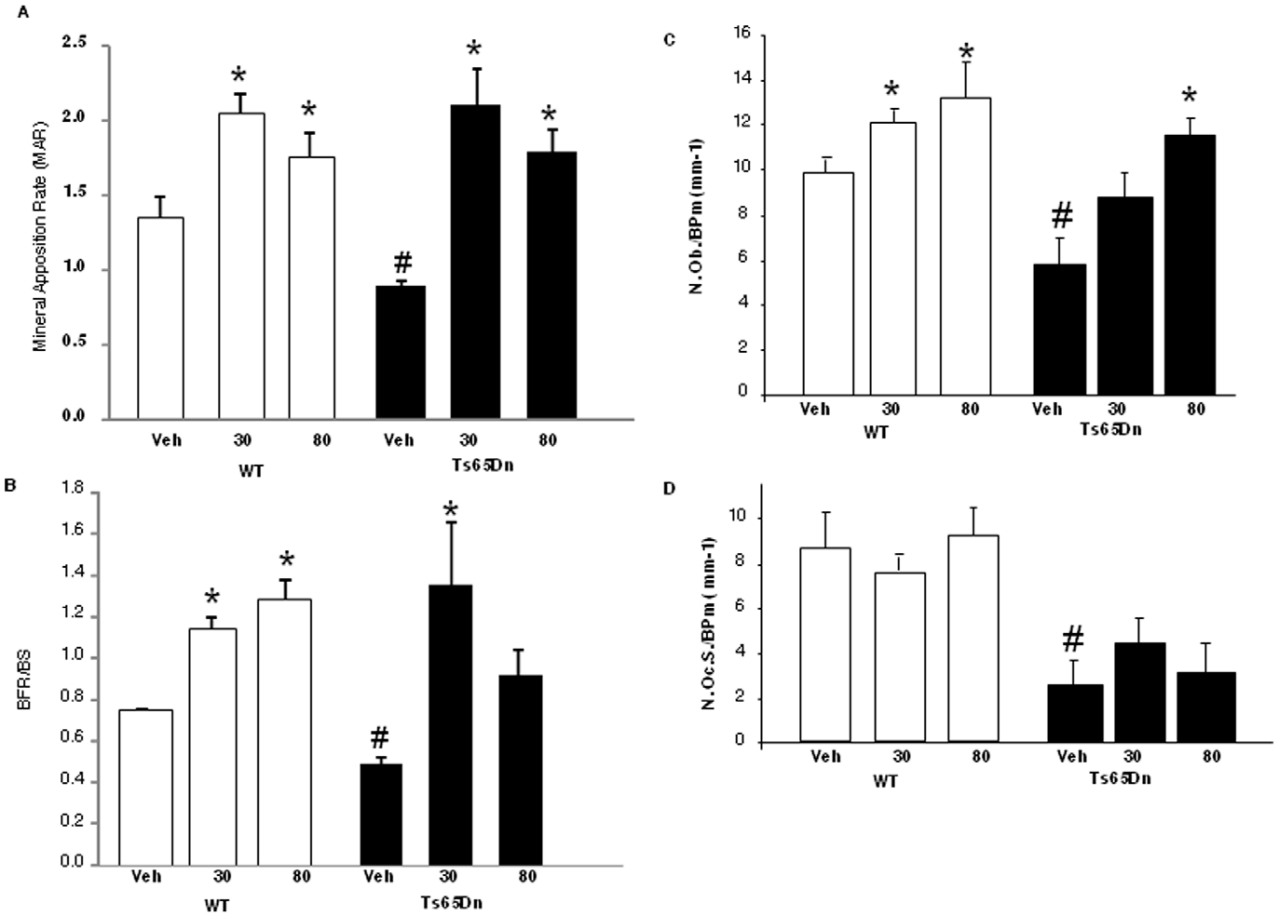
Histomorphometric measurement of bone formation and bone resorption following intermittent PTH in WT and Ts65Dn mice. Male mice (N = 6–8 per group) were given daily injections of vehicle or 30 or 80 ug/kg PTH(1–34) for 4 weeks. (**A**) Mineral apposition rate (MAR) (um^3^/um^2^/day), (**B**) Bone formation rate/bone surface (BFR/BS) (um^3^/um^2^/day), (**C**) Number of osteoblasts/bone perimeter (N.Ob./B.Pm), (**D**) Number of osteoclasts/bone perimeter (N.Oc./B.Pm.) were measured in WT and Ts65Dn mice vehicle or PTH treated (30 or 80 ug/kg/day) (open bars, WT; closed bars, Ts65Dn). *, p<0.05 vs. respective vehicle control; #, p<0.05 vs. WT vehicle.

Mice were treated with daily i.p. injections of 30 or 80 ug/kg PTH(1–34), or vehicle control, for 28 days. PTH increased whole-body BMD in both 3 month old WT and Ts65Dn mice as determined by DXA (Figure 4A). In WT mice, both PTH treatment doses significantly increased whole-body BMD compared with untreated mice. In contrast in Ts65Dn mice, although both PTH doses increased whole body BMD only the 80 ug/kg dose reached statistical significance (Figure 4A). The magnitude of the increase in BMD in Ts65Dn mice was similar to the magnitude of the response in WT mice (Figure 4A). Bone volume fraction (BV/TV) in the tibia was lower in Ts65Dn than age-matched WT mice (Figure 4B). The effect of PTH on the trabecular bone compartment of the tibia (Figure 4B) showed that the effect of PTH on trabecular BMD and BV/TV was similar in both WT and Ts65Dn mice (Figure 4B). As with the whole-body DXA measurements, the PTH effect was significant only at the 80 ug/kg dose in Ts65Dn mice. In both treatment groups, the PTH-stimulated increase in trabecular BV/TV (Figure 5B) was associated with significantly increased Tb.N. and Tb.Th. (Figure 4B), as expected for anabolic PTH therapy.

Intermittent PTH treatment caused an approximately equivalent increase in BV/TV in both WT and Ts65Dn mice (Figure 4B). PTH increased bone volume in both metaphyseal trabecular bone (Figure 5B) as well as diaphyseal cortical bone in both WT and Ts65Dn mice (Figure 4C). The magnitude of the PTH-stimulated increase in BMD and cortical thickness in the femur was similar in both WT and Ts65Dn mice. In both groups PTH treatment increased both endocortical and periosteal surfaces, measured by microCT as a decrease in endocortical circumference and a corresponding increase in periosteal circumference (Figure 4C), with no apparent changes in cortical porosity (data not shown).

The observed increase in trabecular bone mass and volume was mediated by a PTH-stimulated increase in mineral apposition rate and bone formation (Figure 5A, B). Osteoblast number/bone perimeter (N.Ob./BPm) was increased significantly in response to PTH treatment in both WT and Ts65Dn animals, indicating an ability of PTH to overcome the suppression in bone formation induced by trisomy 21 (Figure 5C). In contrast, 4 weeks of intermittent PTH treatment did not increase any osteoclast parameter measured, including osteoclast number per bone perimeter (N.Oc./BPm) in either WT or Ts65Dn mice (Figure 5D).

## Discussion

The present study demonstrated that Ts65Dn DS mice have lower trabecular bone volume and trabecular number and higher trabecular separation in the proximal tibia and distal femur as well as decreased cortical thickness and strength compared with WT littermates. The low bone mass phenotype is consistent with the human DS population that also displays a low BMD due to decreased bone turnover [13]. Histomorphometric analysis of the trabecular bone in Ts65Dn mice revealed a reduction in osteoblast and osteoclast activity and decreased cell numbers on the bone surface, leading to decreased bone turnover markers. These observations result in a significantly lower MAR and BFR in 12 week old Ts65Dn mice, leading to the decreased bone mass and volume observed. Interestingly, histological evaluation also demonstrated the age-related loss of trabecular bone in both WT and Ts65Dn mice, but that the characteristic increase in marrow fat was absent in Ts65Dn. In *ex vivo* bone marrow cultures, the differentiation capacity of osteoblasts and osteoclasts were impaired in Ts65Dn mice, demonstrating the cell intrinsic nature of the bone phenotype, and confirming both mesenchymal and hematopoetic lineages as targets of trisomy 21. Interestingly, and as reported by others [24], Ts65Dn mice also display significant megakaryocyte hyperplasia, supporting our conclusion of a distorted hematopoietic stem cell compartment in these mice. Thus, the low bone mass of Ts65Dn mice phenocopies the low bone mass observed in DS patients and is the result of low bone turnover; characterized by decreased bone formation and bone resorption. These results are important because they confirm that the Ts65Dn mouse is an accurate model for studying the effects of trisomy on the appendicular skeleton [14] and provide a cellular mechanism for the low bone mass and increased fracture risk of DS patients, that is not related to lifestyle differences, as others have suggested [15], [25], [26].

Ts65Dn mice at 3-months and 24-months of age have significant decreases in trabecular and cortical bone. The BV/TV and trabecular number are significantly decreased at both ages. With the decreased number of trabeculae, there was a subsequent significant increase in trabecular spacing. Interestingly, trabecular thickness was not significantly affected, suggesting that the fundamental mechanism controlling the development of trabeculae is intact in trisomic Ts65Dn mice, but the rate at which they are generated is decreased. These results extend those of Blazek [14] who analyzed femurs from 6 week and 16 week old Ts65Dn mice and observed an identical decreased bone mass and normal trabecular thickness phenotype and Parsons [27] who analyzed the presphenoid bone, but not the axial or appendicular skeletal elements from a wide age range of Ts65Dn mice. While the differences in cancellous bone in Ts65Dn mice revealed significant osteopenia, analysis of cortical bone demonstrated decreased mechanical integrity. Geometric parameters of the femur were significantly decreased compared to age matched WT control femurs. These results were supported by three-point bending studies that revealed significantly decreased mechanical strength of the femurs of Ts65Dn mice.

Other DS mouse models have been developed and their bone phenotype investigated [7]. These murine DS models (Ts1Rhr and Ms1Rhr) are trisomic and monosomic, respectively for only a small portion of mouse chromosome 16 (MMU16) and contain only 33 trisomic genes that are orthologs to those on human chromosome 21 [7], compared with approximately 100 in Ts65Dn mice. Ts1Rhr mice were similar to normal mice, suggesting that a larger region of trisomy is necessary to recapitulate the DS bone phenotype. Interestingly, 3 week old Ms1Rhr mice like Ts65Dn have a low bone mass phenotype with reduced cortical bone geometry, decreased trabecular bone volume and connectivity and unchanged Tb.N. In contrast to Ts65Dn mice, Tb.Th was decreased in Ms1Rhr mice, which is presumably associated with the different number of trisomic genes in these mice. Unfortunately, the authors did not measure bone turnover markers or perform cellular histomorphometry, so the cellular mechanism responsible for the bone phenotype is unknown [7]. Given the limited number of trisomic genes in Ms1Rhr it is perhaps not surprising that the complete bone phenotype of Ts65Dn or people with DS is not recapitulated, although the overall decrease in bone mass, bone volume and bone strength is maintained.

The low bone mass phenotype of Ts65Dn male mice like people with DS is profound and is the result of decreased bone turnover. The decreased bone turnover is unexpected, given the hypogonadism and infertility associated with the disorder in humans [28] and in Ts65Dn mice [29]. In fact, numerous published studies have yet to completely elucidate the pathophysiology of the infertility in men with Down syndrome [28], [30], [31], [32]. However, it is clear that males with DS are generally infertile and have significant disruption of one or more levels of the hypothalamic-pituitary-gonadal axis resulting in elevated FSH and LH levels and inconsistent testosterone levels that are often in the low-normal range [28]. Whatever the final cause of the hypogonadism and infertility in males with DS, our current understanding of endocrine regulation of bone turnover would suggest increased, not decreased bone turnover in both humans and mice. As such, elucidation of the mechanism driving the low bone mass and low turnover phenotype of Ts65Dn mice warrants continued investigation.

The defect in both osteoblast and osteoclast differentiation in Ts65Dn mice was observed in both *ex vivo* bone marrow cultures and in the histomorphometric analysis of tibias from 3-month old Ts65Dn mice. In *ex vivo* cultures, both osteoblast and osteoclast differentiation was significantly decreased in Ts65Dn compared with control. *In vivo*, osteoblast number per bone surface, osteoclast number per bone surface and bone formation rate were all significantly decreased. These observations suggest an intrinsic defect in both the mesenchymal and the hematopoetic cell lineages. While the defect in megakaryocyotopoiesis in DS has been well studied in humans and is recapitulated in the Ts65Dn mouse [24], this is the first report of a cellular defect in both osteoblastogenesis and osteoclastogenesis in Ts65Dn mice.

Histological evaluation of the bone marrow confirmed the megakaryocyte hyperplasia as has been reported in Ts65Dn mice and in DS patients [2], [33], as well as a significant and normal age-related decrease in bone mass in Ts65Dn mice. This is particularly interesting given that DS patients have a life expectancy well into the 5^th^ decade of life and are presenting in increasing numbers with low bone mass and fracture [34]. The effect of trisomy on osteoclast and osteoblast development also inhibited the age-related differentiation of adipocytes in the bone marrow. Our findings are in agreement with a pathologic post-mortem examination of vertebral sections from a 49-year old DS female that reported a complete lack of active osteoclasts and a decreased osteoblast number along the trabeculae [35].

Collectively, these observations provide evidence that the low bone mass observed in humans with DS and Ts65Dn mice is due to a cellular defect in both the hematopoietic and mesenchymal lineages, such that aneuploidy’s effects on the available pool of precursor cells decreases their capacity for normal differentiation and function. Thus, fewer cells differentiating into functional osteoblasts and osteoclasts results in the accrual of lower bone mass, which is a mechanism fundamentally distinct from the high bone turnover observed in hypogonadal women and men, where increased numbers and activity of cells leads to low bone mass.

In support of this idea, the Ts65Dn mice demonstrate a lifelong deficit in bone turnover, such that normal peak adult bone mass is never achieved. It is important to note that the serum bone turnover markers of control animals revealed the expected age-dependent increases in both formation and resorption markers, whereas markers in Ts65Dn mice do not increase with age and are consistently lower compared with control throughout the life of the mice. If this temporal difference in bone turnover is recapitulated in humans with DS, it would indicate that low bone turnover is sustained in DS resulting in lower bone accrual and is an area that we are actively investigating. Perhaps most importantly, our studies provide the first data to demonstrate the efficacy of a bone anabolic agent in the treatment of low bone mass in DS and call into question the routine use of bisphosphonates (which decrease bone turnover) in this patient population.

Intermittent PTH therapy increases bone formation by stimulating osteoblast numbers at sites of bone remodeling, where old bone is replaced with new by the concerted efforts of both osteoclasts and osteoblasts [36], [37]. In Ts65Dn mice PTH(1–34) treatment significantly increased bone mass and volume at the highest dose tested (80 ug/kg). PTH stimulated both mineral apposition rate and bone formation rate resulting in increased bone mass, consistent with the reported effects of PTH in the treatment of both post menopausal and glucocorticoid-induced osteoporosis [38], [39], [40]. Interestingly, PTH treatment in both WT and Ts65Dn mice increased bone mass and osteoblastic activity (MAR and BFR) to a similar extent, despite the low BMD and decreased bone turnover status of Ts65Dn mice. The stimulation of bone mass by intermittent PTH treatment demonstrated the efficacy of the intervention in trisomy 21 and supports the idea that PTH or other potential anabolic treatments, such as anti-sclerostin antibody [41], [42], may have utility in DS patients with low BMD and low bone turnover.

Thus, these data provide a clearer understanding of the cellular mechanisms leading to the low bone mass in DS, suggest a new direction for treatment and provide a relevant animal model for continued studies of the skeleton in DS. Given the elevated fracture risk and rate in DS patients our data provide the rationale to consider bone anabolic therapies in DS patients with low bone mass.

## Methods

### Animals and Husbandry

All animal handling and experimentation was performed in accordance with approved University of Arkansas for Medical Sciences (UAMS) institutional guidelines and protocols and approved by the UAMS Institutional Animal Care and Use Committee (IACUC). Ts65Dn male mice and wild type euploid littermate controls were purchased from the Jackson Laboratory (Bar Harbor, ME). Upon arrival they were housed individually with food and water available *ad libitum* and a 12-hr light:dark cycle. Only male mice were evaluated due to the decreased fertility of Ts65Dn male mice and the necessity of Ts65Dn female mice for colony maintenance. Animals were purchased at 6 weeks and aged as required. Some mice were aged and maintained at UAMS for up to 24 months.

For bone phenotyping, animals were sacrificed at 3 months and 24 months and long bones and spines harvested. Some three month old animals were injected with calcein (Sigma, St. Louis, MO) (administered 8 days prior to sacrifice) and alizarin red S (Sigma) or alizarin red complexone (Sigma) (administered 2 days prior to sacrifice), for subsequent determination of dynamic parameters of bone histomorphometry with an inter-label distance of 6 days as described [22]. The BMD of tibia and femur was determined before and after PTH administration with a PIXImus densitometer (GE-Lunar Corp., Madison, WI), as previously described [22]. Synthetic human PTH(1–34) (Creative Peptides, Shirley, NY) was injected daily, i.p., at 30 and 80 ug/kg body weight for 28 days. Weight changes ranged from 2–10% during the course of the experiment in either Ts65Dn or WT control mice and were not significantly different. Vehicle-injected mice of both genotypes served as controls.

### Biochemical Markers

In Ts65Dn and WT mice, serum was extracted from blood drawn at sacrifice and serum procollagen-1 N-terminal peptide (P1NP) and tartrate resistant acid phosphatase (TRAP) measured. P1NP was measured using a commercially available enzyme immunoassay (EIA) (Immunodiagnostic Systems) and TRAP using a TRAP-5b solid phase immunofixed enzyme activity assay (Immunodiagnostic Systems) according to the manufacturer’s protocol.

### Trabecular Bone Assessment by Micro Computed Tomography (CT)

All MicroCT analyses were consistent with current guidelines for the assessment of bone microstructure in rodents using micro-computed tomography [43]. Formalin-fixed tibiae and femora were imaged using a MicroCT 40 (Scanco Medical AG, Bassersdorf, Switzerland) using a 12 µm isotropic voxel size in all dimensions. The region of interest selected for analysis comprised 240 transverse CT slices representing the entire medullary volume extending 1.24 mm distal to the end of the primary spongiosa with a border lying 100 um from the cortex. Three-dimensional reconstructions were created by stacking the regions of interest from each two-dimensional slice and then applying a gray-scale threshold and Gaussian noise filter as described [19] using a consistent and pre-determined threshold with all data acquired at 70 kVp, 114 mA, and 200-ms integration time. Fractional bone volume (bone volume/tissue volume; BV/TV) and architectural properties of trabecular bone (trabecular thickness (Tb.Th., µm), trabecular number (Tb.N., mm-1), and connectivity density (Conn. D, mm-3) were calculated using previously published methods [19].

### Cortical Bone Assessment by MicroCT

The MicroCT slices were segmented into bone and marrow regions by applying a visually chosen, fixed threshold for all samples after smoothing the image with a three-dimensional Gaussian low-pass filter (σ = 0.8, support = 1.0) to remove noise, and a fixed threshold (30% of maximal gray scale value). Femoral cortical geometry was assessed in a 1-mm-long region centered at the femoral midshaft. The outer contour of the bone was found automatically using the built-in manufacturer’s contouring tool. Total area was calculated by counting all voxels within the contour, bone area by counting all voxels that were segmented as bone, and marrow area was calculated as total area - bone area. This calculation was performed on all 25 slices (1 slice = ∼12.5 µm), using the average for the final calculation. The outer and inner perimeter of the cortical midshaft was determined by a three-dimensional triangulation of the bone surface (BS) of the 25 slices, and cortical thickness and other cortical parameters were determined as described [19].

### Histology

After MicroCT analysis, the same tibia and femora, fixed in 10% neutral-buffered formalin, were decalcified in 5% formic acid with agitation until deemed clear by the ammonium oxalate endpoint test [19]. One tibia and one femur were embedded in paraffin, sectioned at 5 um, and stained with hematoxylin and eosin (H&E). The decalcified specimens were then dehydrated through graded ethanol, cleared in methyl salicylate, embedded in paraffin, sectioned (4 µm), and stained with hemotoxylin and eosin (H&E) as described [22].

### Bone Histomorphometry

Specimens for plastic embedding and cancellous bone histomorphometry were harvested at sacrifice and the muscle dissected away before fixation in Mallonig’s fixative as we have described previously [19], [20], [22], [44], [45]. For static histomorphometric analyses, 4–5 um-thick central saggital sections of undecalcified MMA embedded tibiae were stained for TRAP and counterstained with hematoxylin to determine osteoclast numbers and eroded surface, or with Masson’s Trichrome for all other measurements, as defined by Parfitt [46] using Osteomeasure software (Osteometrics, Atlanta, GA). Dynamic measurement of bone formation was performed by injecting mice with 30 mg/kg of calcein 8-days before sacrifice and alizarin red S (15 mg/kg) or alizarin red complexone (18 mg/kg) 2-days before sacrifice [39], [41]. The proportion of single and double labeled perimeters and the interlabel distance between the fluorophores were measured in the cancellous bone of unstained sections adjacent to those used for static measurements [19], [20], [22], [45]. All cancellous bone measurements were made within the area defined by 700–1500 um distal to the growth plate-metaphyseal junction of the proximal tibia and 150 um away from either endocortical surface. A minimum of eight 20x fields in the proximal tibia were evaluated. Similarly, static cellular measurements of osteoblasts and osteoclasts were obtained as described [19], [20], [22]. Osteoclasts were enumerated in sections stained for TRAP, with osteoclasts identified as TRAP multinucleated cells, adjacent to bone. Osteoblasts were identified as layers of cuboidal cells aligned along a bone surface. All cellular and dynamic parameters are reported using the terminology recommended by the Histomorphometry Nomenclature Committee of American Society for Bone and Mineral Research [46] and as we have described [19], [20].

### Whole Femur Mechanical Testing

Mechanical testing was performed to determine bone strength of frozen Ts65Dn intact femora (3 month and 24 month), using standard three-point bending as we have described [19]. The thawed femurs were soaked in saline for 1 hour before testing to ensure hydration and then tested at room temperature using a servohydraulic-testing machine MTS 858 Bionex test systems load frame (MTS, Eden Prairie, MN) with computer control, data logging, and calculations of load to failure using TestWorks version 4.0 (MTS). Femurs were placed anterior side down on two horizontal supports spaced 7 mm apart; the central loading point contacted the posterior surface of the diaphysis at the midpoint of the bone length. The loading point was displaced downward (transverse to the long axis of the bone) at 0.1 mm/second until failure, generating bending in the anteroposterior plane. Load displacement data were recorded at 100 Hz (TestWorks 4.0, MTS), and test curves were analyzed to determine measures of whole-bone strength, primarily peak load and stiffness as we have described [19]. Load-to-failure was reported as the load after a 2% drop from peak load.

### Ex vivo Marrow Cultures

Bone marrow cells were harvested from femurs for osteoclastogenic culture as previously described [19], [21]. Briefly, cells were flushed from femurs, washed, and cultured in 24-well plates (Becton Dickinson Labware) at a density of 1×10^6^ cells per well in α-minimal essential medium (α-MEM), supplemented with 15% fetal calf serum, and 10^−8^ M 1,25-dihydroxyvitamin D3 (1,25(OH)2D3 in quadruplicate wells per treatment. Cells were fed every 3 days with half-volumes of medium, until day 14, when cells were fixed and stained with tartrate resistant acid phosphatase (TRAP) to facilitate determination of the number of TRAP-positive multinucleated (3 or more nuclei) cells formed per well.

For osteoblastogenesis, bone marrow cells were harvested from femurs and seeded in triplicate at 2.5×10^5^ cells/cm^2^ on six-well tissue culture plates (Becton Dickinson Labware) osteoblast medium (α-MEM +15%FBS containing 10 mM BGP and 50 µM ascorbic acid). The total number of mesenchymal progenitors recruited into the osteoblastic lineage was measured by alkaline phosphatase-positive staining for colony forming unit-fibroblast (CFU-F) at day 10. Replicate cultures were fed every 3 days with half-volumes of medium, until day 28, when cells were fixed and minerals stained with Alizarin Red to facilitate determination of the number of bone nodules representing CFU-osteoblastic (CFU-OB) formed per well [19], [21].

### Statistical Analyses

All data were analyzed by either Student’s t-test or one way ANOVA with post-hoc analyses by Bonferroni post-hoc test as appropriate. Parametric data are presented at mean ± SEM. Differences with P values <0.05 were considered statistically significant and are reported as such. The SigmaStat (SPSS Science) software package was used for all statistical analysis.

## Supporting Information

Figure S1 **Age-related bone phenotype in Ts65Dn mice.** (**A**) Micro CT reconstructions of proximal tibia and femur from 3-month (left column) and 24-month (right column) old WT (top) and Ts65Dn (bottom) mice. Low bone volume and cortical thinning is evident. (**B**). Paraffin-embedded decalcified histological sections of proximal tibia from 3-month (left column) and 24-month (right column) old WT (top) and Ts65Dn (bottom) mice stained with H&E. The expected age-related decrease in trabecular bone is observed in both WT (top) and Ts65Dn (bottom), however, the expected increase in marrow fat is observed in WT (top) but not Ts65Dn (bottom). In contrast, Ts65Dn mice reveal the reported elevation in marrow megakaryocyte number [24] that increases with age (bottom), that is not observed in WT control mice (top). Original magnification 4X
(TIF)

Figure S2 **Decreased cortical bone parameters of 24-month old Ts65Dn mice.** Determination of femoral midshaft cortical parameters from Micro CT reconstructions of mid shaft femur shows significant decreases compared to WT in (**A**) cortical cross sectional area, (**B**) total cross sectional area, (**C**) periosteal perimeter, (**D**) Peak load (load tolerated at the breaking point adjusted for bone size), (**E**) Stiffness (deformation tolerated before breaking). Open bars WT; solid bars Ts65Dn. *, p<0.05 vs. WT control.
(TIF)
