# Supplementary figures and images for: Low Bone Turnover and Low BMD in Down Syndrome: Effect of Intermittent PTH Treatment
Source: PLoS One. 2012 Aug 14;7(8):e42967. doi: 10.1371/journal.pone.0042967 (PMC3419249; doi:10.1371/journal.pone.0042967)

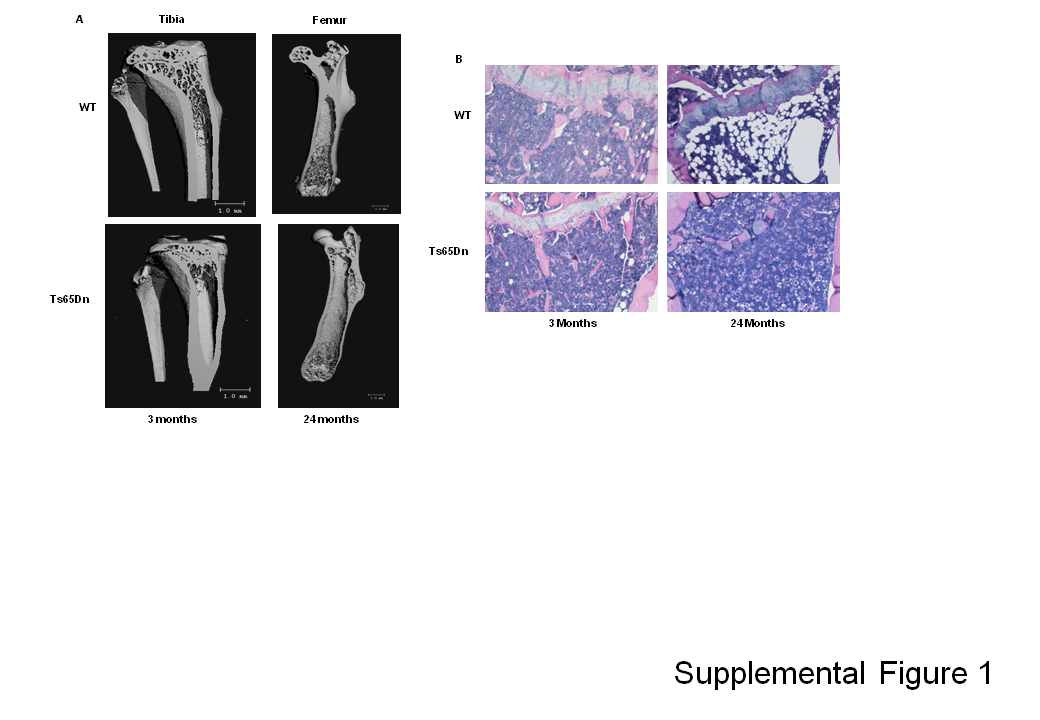

Supplement: Figure S1 — Age-related bone phenotype in Ts65Dn mice. (A) Micro CT reconstructions of proximal tibia and femur from 3-month (left column) and 24-month (right column) old WT (top) and Ts65Dn (bottom) mice. Low bone volume and cortical thinning is evident. (B). Paraffin-embedded decalcified histological sections of proximal tibia from 3-month (left column) and 24-month (right column) old WT (top) and Ts65Dn (bottom) mice stained with H&E. The expected age-related decrease in trabecular bone is observed in both WT (top) and Ts65Dn (bottom), however, the expected increase in marrow fat is observed in WT (top) but not Ts65Dn (bottom). In contrast, Ts65Dn mice reveal the reported elevation in marrow megakaryocyte number [24] that increases with age (bottom), that is not observed in WT control mice (top). Original magnification 4X (TIF) [file pone.0042967.s001.tif]

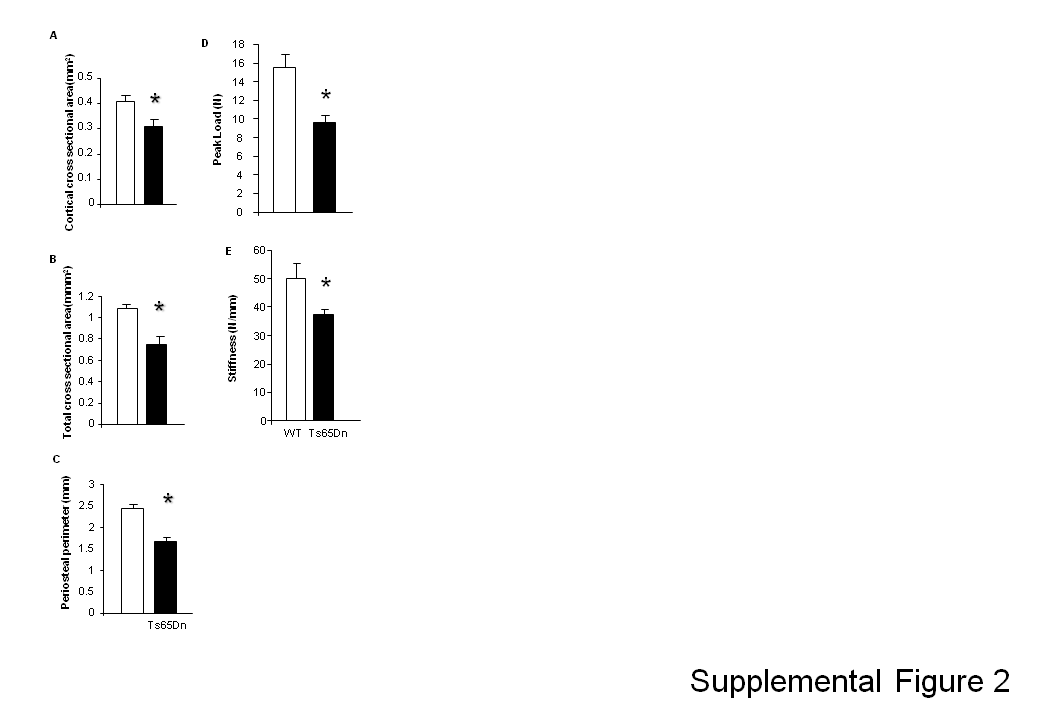

Supplement: Figure S2 — Decreased cortical bone parameters of 24-month old Ts65Dn mice. Determination of femoral midshaft cortical parameters from Micro CT reconstructions of mid shaft femur shows significant decreases compared to WT in (A) cortical cross sectional area, (B) total cross sectional area, (C) periosteal perimeter, (D) Peak load (load tolerated at the breaking point adjusted for bone size), (E) Stiffness (deformation tolerated before breaking). Open bars WT; solid bars Ts65Dn. *, p<0.05 vs. WT control. (TIF) [file pone.0042967.s002.tif]
